# High-dose intravenous immunoglobulins reduce nerve macrophage infiltration and the severity of bortezomib-induced peripheral neurotoxicity in rats

**DOI:** 10.1186/s12974-018-1270-x

**Published:** 2018-08-21

**Authors:** Cristina Meregalli, Ivan Marjanovic, Carla Scali, Laura Monza, Nadia Spinoni, Cristina Galliani, Rinaldo Brivio, Alessia Chiorazzi, Elisa Ballarini, Virginia Rodriguez-Menendez, Valentina Alda Carozzi, Paola Alberti, Giulia Fumagalli, Eleonora Pozzi, Annalisa Canta, Marina Quartu, Chiara Briani, Norberto Oggioni, Paola Marmiroli, Guido Cavaletti

**Affiliations:** 1 School of Medicine and Surgery, Experimental Neurology Unit and Milan Center for Neuroscience, University of Milano-Bicocca, via Cadore 48, 20900 Monza, MB Italy; 2 Kedrion S.p.A, Loc. Ai Conti, Castelvecchio Pascoli, Lucca, Italy; 3 PhD program in Translational and Molecular Medicine (Dimet), University of Milano-Bicocca, Monza, Italy; 4 Clinical Chemistry Laboratory, S. Gerardo Hospital, Monza, Italy; 5 Young Against Pain group, Parma, Italy; 6 PhD program in Neuroscience, University of Milano-Bicocca, Monza, Italy; 7 Department of Biomedical Sciences, University of Cagliari, Cittadella Universitaria, Monserrato, Italy; 8 Department of Neuroscience, Neurology Unit, University of Padova, Padova, Italy

**Keywords:** Bortezomib, Peripheral neurotoxicity, Allodynia, Human intravenous immunoglobulin (IVIG), Neuroinflammation

## Abstract

**Background:**

Chemotherapy-induced peripheral neurotoxicity (CIPN) is a severe adverse effect in patients receiving antitumor agents, and no effective treatment is available. Although the mechanisms responsible for the development of CIPN are poorly understood, recent findings make neuroinflammation an attractive target to be investigated, particularly when neuropathic pain is a prominent feature such as after bortezomib administration.

The aim of our study was to evaluate the effect of intravenous immunoglobulins (IVIg) delivery in chronic CIPN. The related neuro-immune aspects were investigated in a well-characterized rat model of bortezomib-induced peripheral neurotoxicity (BIPN).

**Methods:**

After determination of a suitable schedule based on a preliminary pharmacokinetic pilot study, female Wistar rats were treated with IVIg 1 g/kg every 2 weeks. IVIg treatment was started at the beginning of bortezomib administration (“preventive” schedule), or once BIPN was already ensued after 4 weeks of treatment (“therapeutic” schedule). Neurophysiological and behavioral studies were performed to assess the extent of painful peripheral neurotoxicity induced by bortezomib, and these functional assessments were completed by pathologic examination of peripheral nerves and intraepidermal nerve fiber quantification (IENF). The role of the innate immune response in BIPN was investigated by immunochemistry characterization of macrophage infiltration in peripheral nerves.

**Results:**

Both schedules of IVIg administration were able to significantly reduce bortezomib-induced heat and mechanical allodynia. Although these changes were not evidenced at the neurophysiological examination of peripheral nerves, they behavioral effects were paralleled in the animals treated with the preventive schedule by reduced axonopathy in peripheral nerves and significant protection from loss of IENF. Moreover, IVIg administration was very effective in reducing infiltration in peripheral nerves of macrophages with the M1, pro-inflammatory phenotype.

**Conclusion:**

Our results suggest a prominent role of neuroinflammation in BIPN and that IVIg might be considered as a possible safe and effective therapeutic option preventing M1 macrophage infiltration. However, since neuropathic pain is frequent also in other CIPN types, it also indicates the need for further investigation in other forms of CIPN.

## Background

Over the last decade, chemotherapy-induced peripheral neurotoxicity (CIPN) has emerged as a severe adverse effect in patients receiving antitumor agents, such as platinum compounds, antitubulins, thalidomide, and bortezomib. Patients who develop CIPN frequently complain of glove-and-stocking sensory loss, paresthesia, often associated with neuropathic pain, which severely affects their quality of life [1, 2]. Despite extensive research, there are still no approved drugs for treatment or prevention of CIPN.

Although the mechanisms responsible for the development of CIPN are poorly understood, recent findings make neuroinflammation an attractive target to be investigated [3–5]. Altered immunological response in cancer patients undergoing neurotoxic chemotherapy has already been demonstrated. Paclitaxel (PTX) induces systemic Treg cell impairment [6], and elevated serum pro-inflammatory cytokines levels have been detected in breast cancer patients treated with taxanes, which are also responsible for enhanced activity of peripheral blood natural killer cells and activation of cytotoxic cells through lymphocytes [7]. Similarly, acute immune response after oxaliplatin treatment in colorectal patients has been associated with increased inflammatory cytokine levels [8].

Regarding pathological mechanisms contributing to CIPN, preclinical data have demonstrated the involvement of both innate and adaptive immune responses, as well as effects of anticancer drugs on peripheral and central neuronal accessory cells, including glial cells (satellite cells), Schwann cells, astrocytes, and microglia [9].

Therefore, it is reasonable to hypothesize that the pathogenic mechanisms underlying CIPN could share common features with changes in systemic immunological response induced by antineoplastic drugs.

Despite current evidence is still insufficient to fully explain the role of neuroinflammation in CIPN, blockade of proinflammatory signaling by interleukin-1 receptor antagonist and antibodies anti-tumor necrosis factor alpha have been reported to attenuate CIPN symptoms in rodent studies [10–12]. Moreover, treatment with anti-inflammatory mediators including interleukin-10 and regulatory T cells allowed recovering from chemotherapy-induced neuropathic pain in rodents [10, 13, 14].

Bortezomib-induced neurotoxicity (BIPN) is peculiar among the different types of CIPN since it is extremely painful. Despite evidence of bortezomib off-target effects (i.e., non directed to inhibit proteasomal activity), which might be related to its neurotoxicity through increased tubulin polymerization in dorsal root ganglia (DRG) neurons and peripheral nerves, the pathogenesis of BIPN is still undetermined, and current treatment inadequate.

High-dose intravenous immunoglobulins (IVIg) are widely used as immunomodulatory therapy in several immune-mediated diseases (including inflammatory neuropathies) and acute inflammatory conditions [15, 16]. Moreover, a clinical study reported IVIg-induced improvement in a small series of BIPN patients [17]. The aim of our study was first to assess the optimal IVIg schedule in rats and, soon thereafter, to test with a multimodal approach the effect of IVIg in a well-established rat model of BIPN [18].

## Methods

### Ethics

The experiments were compliant with the ethics guidelines described in national (D L.vo 26/2014, Gazzetta Ufficiale della Repubblica Italiana) and international laws (European Union Directive 2010/63/EU: Guide for the Care and Use of Laboratory Animals, US National Research Council, 1996). All procedures were conducted in accordance with protocols approved by the Ethics Committee of the University of Milan Bicocca (no. 0051126).

### Pharmacokinetic (PK) preliminary study

**Fig. 1:**
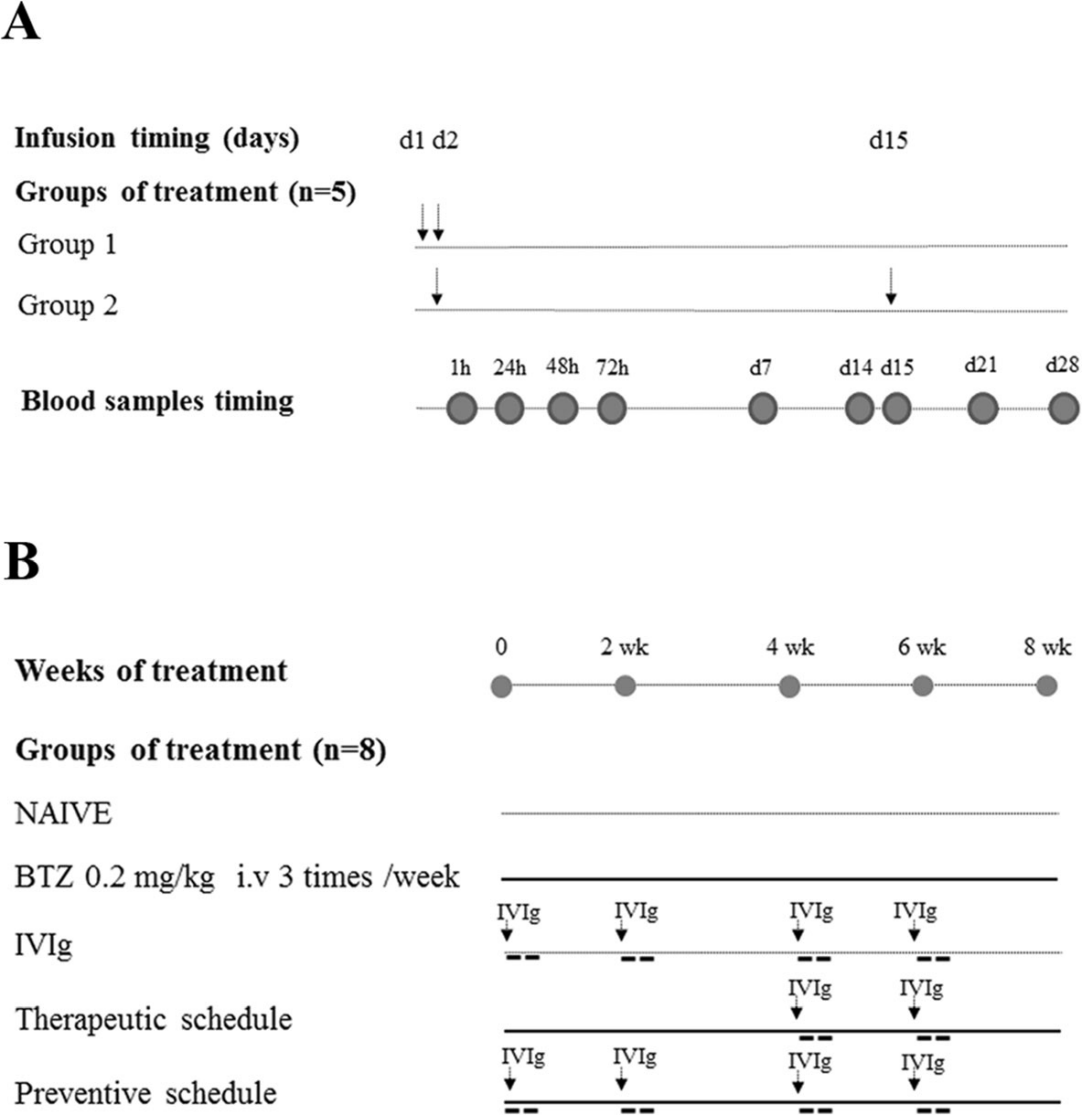
Flow chart of pharmacokinetic pilot study (**a**) and IVIg effects on BIPN experiment (**b**). Arrows indicate each intravenous administration (I.V). Abbreviation: BTZ bortezomib, CTRL control

In order to select a suitable schedule of IVIg to be used for animal studies, we performed a pharmacokinetic study of the immunoglobulins for clinical use (Ig VENA 50 g/l solution for infusion, Kedrion S.p.A, Italy). PK values were obtained from two groups of five female Wistar rats each (175–200 g, Envigo, Udine, Italy) after the administration of 1 g/kg/day over two consecutive days (group 1) or the same dose 1 day and after 2 weeks (group 2). The administration volume was 4 ml, and the infusion time was 10 min (as represented in Fig. 1a).

Blood was collected via the tail vein at days 1, 2, 3, 7, 14, and 28 to determine serum IVIg concentration. After centrifugation at 3500 rpm for 15 min at 20 °C, serum samples were collected and analyzed through automatic nephelometric method, IMMAGE 800 (Beckman Coulter, USA). IVIg serum concentration-time data were further evaluated by means of non-compartmental analysis using the PK software Analyst™ 6.1 (Applied BioSystems, CA, USA). The pharmacokinetic parameters C_max_, Tmax, MRT 0-_inf_, and AUC (0-t) were calculated.

### IVIg effects on BIPN

#### Experimental protocol

The experiment was designed to minimize the number of animals based on pre-study power analysis indicating that eight animals/group were sufficient to achieve significant results using the nerve conduction velocity (NCV) as primary endpoint (power = 80%, α = 5%). Female Wistar rats (175–200 g, Envigo, Udine, Italy) were randomized for the study, and their clinical conditions were monitored daily; the rats were housed under standard conditions at 20 ± 2 °C under a reversed 12 h/12 h light/dark cycle with food and water ad libitum. Body weight was measured before each drug treatment for dose adjustment. Part of the animals were sacrificed under deep anesthesia after 3 weeks of treatment and at the end of the treatment period with BTZ (eighth week) for tissues sampling (see below).

#### Induction of BIPN

To induce the development of BIPN, bortezomib (LC Laboratories, Woburn, MA) was dissolved in 5% Tween 80, 5% ethanol, and 100% and 90% sterile saline. Ig VENA 50 g/l solution for infusion (Kedrion S.p.A, Italy) was used as human IVIg preparation, at the daily dose of 1 g/kg that is the range of human doses for the chronic treatment of inflammatory neuropathy [19]. Animals were randomized into five experimental groups, and all drug treatments were performed between 09.00 a.m. to 11.00 a.m. One group was left untreated (CTRL, *n* = 8); one group was treated with bortezomib IV 0.2 mg/kg, three times/week for 8 weeks (BTZ, *n* = 8); one group was treated with IVIg 1 g/kg administered by infusion every 2 weeks for 8 weeks (IVIg, *n* = 8); one group was co-treated with bortezomib IV 0.2 mg/kg, three times/week for 8 weeks and IVIg 1 g/kg every 2 weeks starting after 4 weeks of BTZ administration (subsequently indicated as “therapeutic” schedule, *n* = 8); one group was co-treated with bortezomib IV 0.2 mg/kg, three times/week for 8 weeks and IVIg 1 g/kg every 2 weeks starting from bortezomib administration (subsequently indicated as “preventive” schedule, *n* = 8) as reported in the Fig. 1b. The control group was left untreated since the neurotoxic effect of bortezomib’s vehicle can be excluded, as reported previously [20].

#### Electrophysiological studies

To evaluate the onset of BIPN, we performed electrophysiological recordings with an electromyography apparatus (Myto2 ABN Neuro, Firenze, Italy). Being BIPN, a length-dependent sensory polyneuropathy [21], we tested two sensory nerves (caudal and digital nerves) as technically feasible to detect neurophysiological alterations. All recordings were performed with subdermal Ambu Neuroline EEG needles (Ambu A/S, Ballerup, Denmark). Caudal nerve was recorded orthodromically: recording cathode and anode were placed at 6 and 5 cm from the tip of the tail, the ground electrode at 2.5 cm from it, and stimulating anode and cathode respectively at 2 and 1 cm. Digital nerve was also studied orthodromically: recording cathode and anode were placed at the base and at the tip of the fourth toe of the hindlimb, respectively, the ground electrode subcutaneously in the sole, and stimulating anode and cathode at the ankle and subcutaneously near the patellar bone, respectively. Intensity, duration, and frequency of stimulation were set up in order to obtain supramaximal results. Filters were kept between 20 and 3 KHz. Animals were kept under deep isoflurane anesthesia, and body temperature was kept constant at 34.5 ± 0.5 °C for the whole procedure.

#### Mechanical and heat threshold determinations

For evaluating neuropathic pain, withdrawal threshold of pain was determined by dynamic test in accordance with a previous established method [18]. Animals were acclimated to the corresponding behavioral test environments, and baseline responses were measured before drug administration.

The occurrence of neuropathic signs was monitored after 3, 4, 5, 7, and 8 weeks of treatment (mechanical threshold) and at 5, 7, and 8 weeks (heat threshold). Mechanical paw withdrawal threshold was assessed using a Dynamic Aesthesiometer Test apparatus (Ugo Basile Biological Instruments, Comerio, Italy). Briefly, *n* = 8 rats/group were placed in a compartment with a wire mesh bottom, and a metal filament was applied to the plantar surface of a hind paw with a progressive increasing puncture pressure, reaching up to 50 g within 20 s. The sensory threshold was recorded automatically, and the mechanical sensitivity was determined by calculating the mean value of six repeated applications. Mechanical measurements were assessed in the morning by a single experimenter (A.C) who was blind to the treatment groups.

Two hours after dynamic test evaluation, withdrawal latency to an infrared heat stimulus was determined using a Plantar Test apparatus (Ugo Basile Biological Instruments, Comerio, Italy) by a single experimenter (L.M) who was blind to the treatment groups. *n* = 8 rats/group were placed in a transparent plastic cage on an elevated plexiglass mesh table. After habituation, a movable infrared radiant heat source (IR 50 mV/cm^2^) was placed directly under the plantar surface on the hind paw, and the time to hind paw withdrawal was monitored (withdrawal latency). The mean of four repeated trials was used for data analysis.

#### Skin biopsy

To detect a damage of the peripheral small nerve fibers, an analysis of intraepidermal nerve fiber (IENF) density has been performed on skin samples of three animals/group collected at sacrifice [22]. Briefly, the skin of the hind paw footpad, 20-μm-thick sections, were prepared and immunostained with rabbit polyclonal anti-protein gene product 9.5 antibodies (PGP 9.5; GeneTex, Irvine, CA, USA) in combination with biotinylated anti-rabbit IgG and Vector SG substrate kit peroxidase (Vector Laboratories, Burlingame, CA) using a free-floating protocol. The IENF density was determined as the number of PGP 9.5-positive fibers per epidermal length by the same blinded examiner (AC).

#### Histopathological analysis

After the third and the eighth weeks from the beginning of the study, all of the animals were sacrificed and caudal and sciatic nerves were collected and processed for morphological and morphometrical analysis according to previously reported protocols [22]. Briefly, caudal and sciatic nerves were immersion-fixed in 3% glutaraldehyde and, subsequently, were osmicated, dehydrated, and embedded in epoxy resin. Specimens were cut, and 1-μm-thick semithin sections were stained with toluidine blue and examined with a Nikon Eclipse E200 light microscope (Nikon Europe B.V, Amsterdam, The Netherlands). In particular, for morphological analysis, × 60 pictures of three randomly selected different fields of each nerve section were taken using a Nikon Eclipse E200 light microscope (Nikon Europe B.V, Amsterdam, The Netherlands). One nerve section per animal from three animals per group of treatment underwent morphometric analysis using an automatic image analyzer compiled by Immagini e Computer SNC (Milan, Italy). Approximately 800–1000 fibers per group were measured by a researcher (AC) who was blind to the treatment groups, and data were then analyzed with GraphPad Prism statistical package (GraphPad Software, San Diego, CA). Axons with evident myelin damage were not included in the measurements.

#### Immunohistochemical (IHC) characterization of the infiltrating cells

To investigate the macrophage infiltration in peripheral nerves, sciatic and caudal nerves of three animals per group were dissected and sacrificed, fixed in 10% formalin overnight (o/n), paraffin embedded, and 3-μm-thick slices were cut with a Leica RM2265 microtome (Microsystems GmbH, Wetzlar, Germany). Immunohistochemistry was performed using anti-CD68 antibody (CD68 Biorad MCA341GA, Segrate Milan, Italy) to detect macrophage infiltrating cells, anti-iNOS antibody (Biorbyt orb13614, Cambridge, UK), and anti-ARG1 antibody (Biorbyt orb394005, Cambridge, UK) to discriminate M1 (pro-inflammatory) from M2 (anti-inflammatory) macrophages. Paraffin sections were deparaffinized with xylene, rehydrated and heated in a steamer for 20 min (1 mM EDTA pH 8 or 10 mM Citrate Buffer pH 5) to retrieve antigens. Endogenous peroxidase activity was quenched by incubation in 3% H_2_O_2_ for 10 min at RT. The slides were washed in PBS and incubated in blocking solution (5% NGS or 3% BSA) for 1 h at RT. Then, the sections were incubated with anti-CD68 antibody (1:300 in 1% NGS), anti-ARG1 (1:50 in 1% BSA), or anti-iNOS (1:500 in 1% BSA) o/n at 4 °C. The following day, the slides were washed and incubated with secondary anti-mouse (1:200, Millipore, Burlington, MA) or anti-rabbit antibody (1:200, Perkin Elmer, Milan, Italy) for 1 h at RT. The antigen-antibody complex was visualized by incubating the sections with 3,3′-diaminobenzidine hydrochloride (Sigma, St. Louis, MO) dissolved in PBS with 10 μl of 3% H_2_O_2_. Negative controls were incubated only with the secondary antibody. Semi-quantitative assessment of macrophage infiltration in the caudal nerve of animals sacrificed after 3 and 8 weeks from the beginning of the experiment was performed by the same blinded examiner (E.B) by scoring + for a scarce presence of inflammation (1–5 macrophages/section); ++ for a more robust infiltration (5–30 macrophages/section); and +++ for a very high infiltration (> 30 macrophages/section).

#### Statistical analyses

The differences in neurophysiological parameters, behavioral tests, and IENF density were statistically evaluated using the analysis of variance (ANOVA) followed by Dunnett’s post hoc tests versus bortezomib experimental group (significance level set at *p* < 0.05).

## Results

### PK pilot study

The administration of IVIg was well tolerated, and no animals showed signs of distress or died during the study. Physiological increase in body weight was observed using both treatment schedules of IVIg administration (data not shown).

**Fig. 2:**
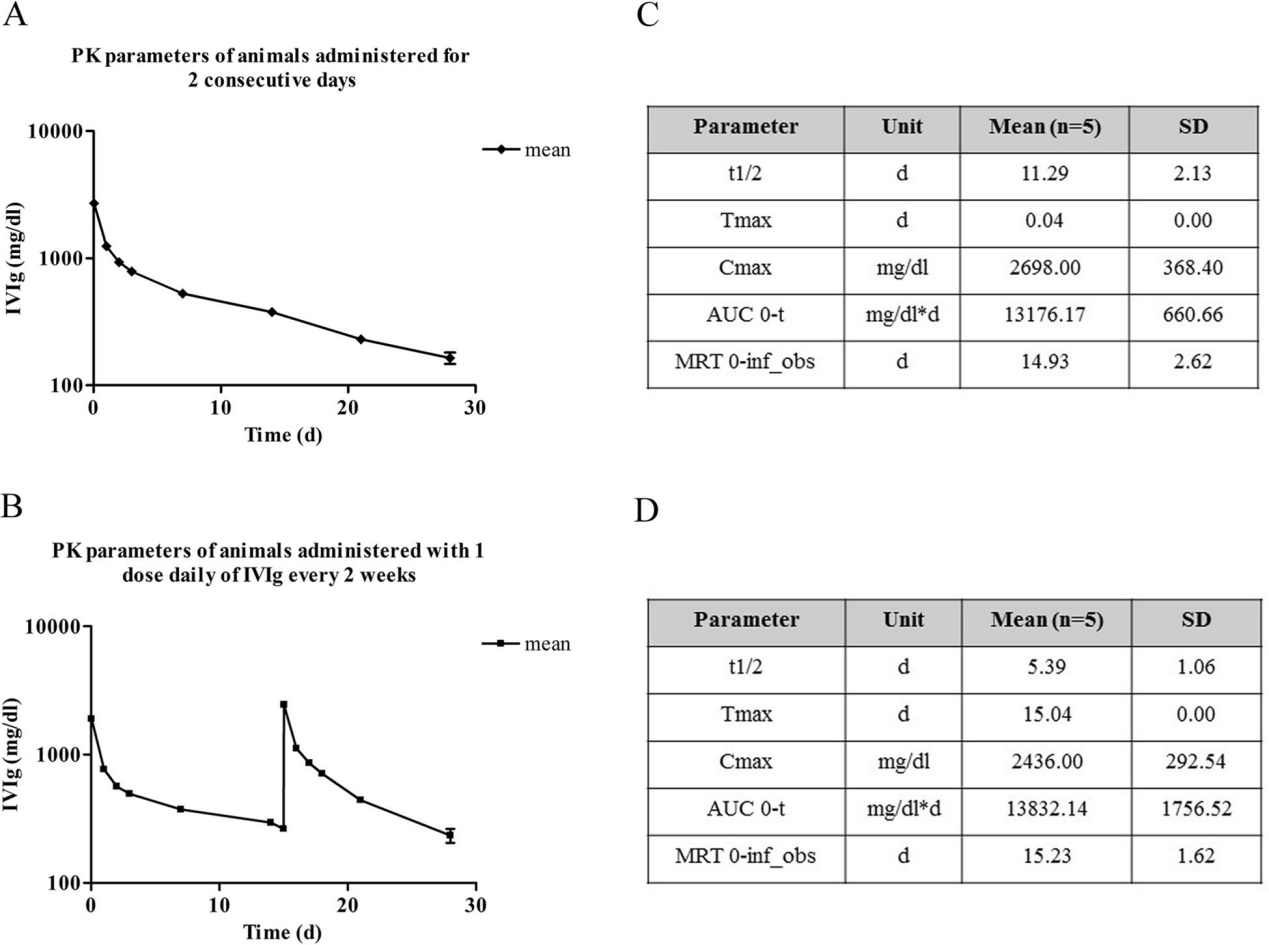
Curves and PK parameters of pilot study. Data obtained from animals treated with IVIg for two consecutive days (**a** and **b**) or with one dose daily every 2 weeks (**c** and **d**). Tmax the time to Cmax, Cmax peak plasma concentration, AUC area under the curve, MRT mean residence time

Serum concentrations of IVIg were measured in each dose group over the entire sampling period, i.e., until 28 days post dose, in all animals. Small difference was observed between the two infusion schedules, but the kinetic curves showed that both schedules allowed keeping levels of IVIg higher than 350 mg/dl for 2 weeks after IVIg administrations (Fig. 2a, c).

The peak plasma concentration (C_max_) and the area under the curve (AUC) values were very similar in both schedule of treatments, while t1/2 was duplicated when IVIg were administered using the two consecutive days schedule (Fig. 2b, d). These results allowed selecting the single daily dose every 2 weeks (days 1 and 15) schedules as the most suitable for the following part of the experiment.

### IVIg effects on BIPN

#### Safety and tolerability of IVIg on body weight measurements

The administration of IVIg was well tolerated by the animals of all groups, and no significant difference in weight gain was observed between groups (data not shown). One animal co-treated with the therapeutic schedule died after the twentieth administration of BTZ, but it was impossible to establish an evident link between death and any treatment. The IVIg delivery resulted in a marked and rapid increase of immunoglobulin serum concentration (2000–3000 mg/dl), followed by a steady decrease after 2 weeks which was in line with the data obtained in the preliminary PK study (360–390 mg/dl).

#### IVIg had no effect on BTZ-induced neurophysiological changes

**Fig. 3:**
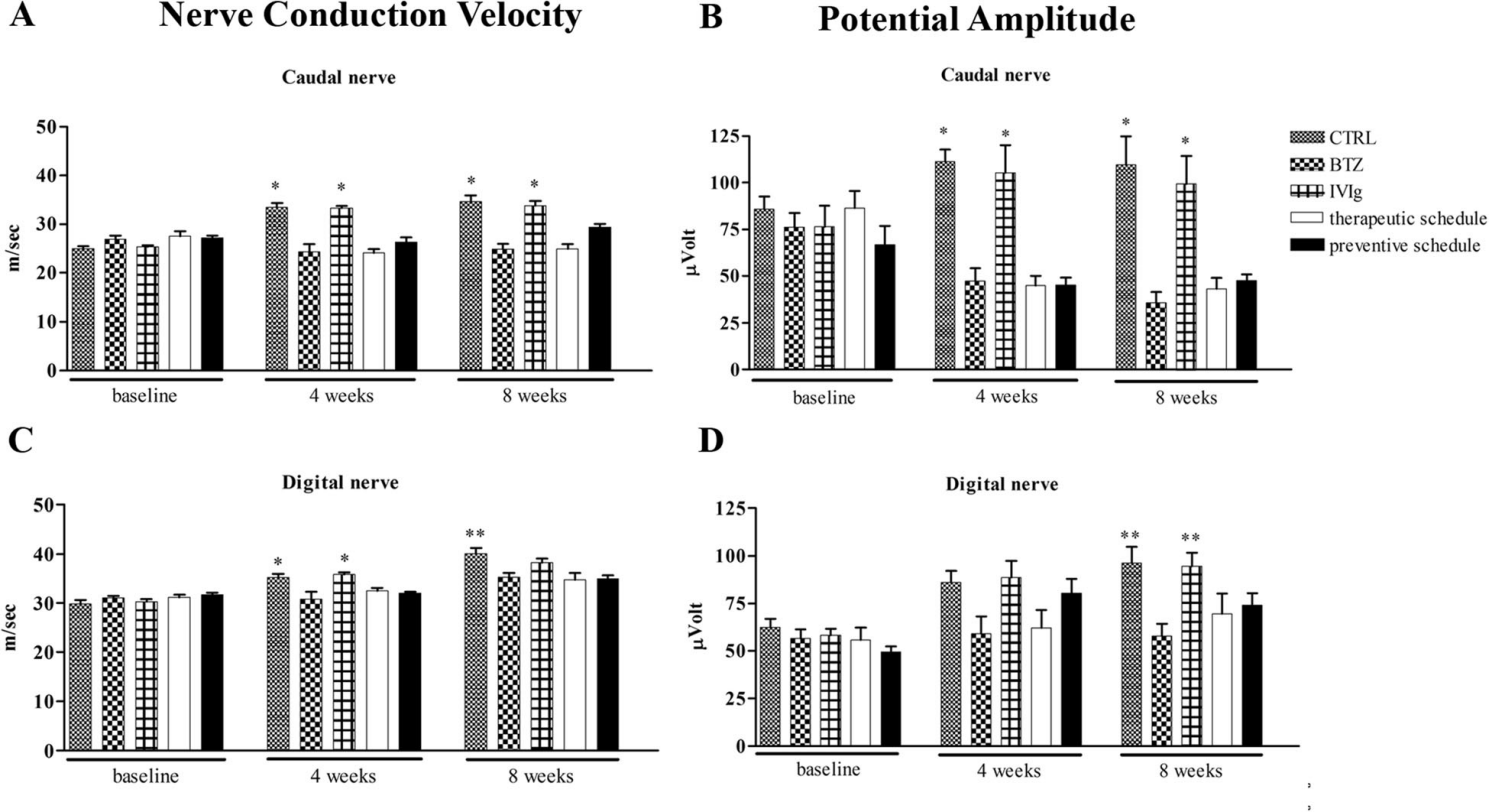
Neurophysiological assessments along the study. The graphs show the development of peripheral neuropathy induced by BTZ already after 4 weeks of treatment; co-treatment with IVIg was not able to prevent BTZ-induced neurophysiological changes. The top panels show (**a**) caudal NCV and (**b**) caudal nerve amplitude; while the bottom panels reproduce (**c**) digital NCV and (**d**) digital nerve amplitude. *p < 0.01 vs BTZ; **p < 0.05 vs BTZ (mean ± SEM)

After 4 weeks of treatment and at the end of the experiment, all BTZ-treated groups had a significant difference in caudal NCV and amplitude if compared both with CTRL and IVIg group (*p* < 0.01 CTRL and IVIg vs BTZ, Fig. 3a, b).

In the digital nerve, after 4 weeks of treatment, NCV was significantly reduced in all BTZ-treated groups vs both CTRL and IVIg groups (*p* < 0.01 CTRL and IVIg vs BTZ, Fig. 3b), while no significant changes in amplitude were observed. At the end of treatment, all BTZ-treated groups showed a significant decrease in digital NCV when compared to CTRL (*p* < 0.05 all BTZ groups vs CTRL Fig. 3c), and a significant decrease in digital amplitude vs both CTRL and IVIg group (*p* < 0.001 CTRL and IVIg vs BTZ Fig. 3d).

#### IVIg administration reduces BTZ-induced mechanical allodynia

**Fig. 4:**
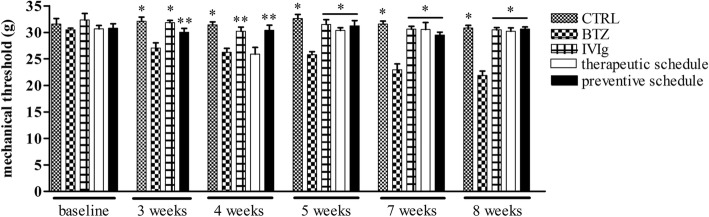
IVIg prevents mechanical allodynia induced by BTZ. Time course of paw withdrawal thresholds in the different groups. Until week 3 from the beginning of the study, rats eventually belonging to the therapeutic schedule group are reported together with the animals of the BTZ group. At the determination performed after 4 weeks, the animals of the therapeutic group had not yet received the first IVIg infusion. Data are reported as mean ± SEM. *p < 0.01 vs BTZ; **p < 0.05 vs BTZ

Already after 3 weeks of treatment, animals treated with BTZ alone showed a significant decrease in the latency to withdrawal at the Dynamic test vs CTRL, IVIg and preventive schedule groups animals (*p* < 0.01 CTRL and IVIg vs BTZ and *p* < 0.05 preventive schedule vs BTZ). This observation was confirmed after 4 weeks of treatment (Fig. 4), when IVIg were started in the therapeutic setting (*p* < 0.01 CTRL vs BTZ and *p* < 0.05 IVIg and preventive schedule vs BTZ).

At the fifth week till the end of the study (eigth week), only animals in the BTZ group showed mechanical allodynia with a reduction in the latency to withdrawal; remarkably, both therapeutic and preventive groups showed a protection from BTZ-induced allodynia if compared to BTZ (*p* < 0.01 all groups vs BTZ) (Fig. 4).

#### IVIg protects from BTZ-induced heat hyperalgesia

After 5 weeks from the beginning of the study, the groups treated with BTZ-alone or together with IVIg showed the development of heat hyperalgesia (CTRL *p* < 0.01 CTRL, IVIg, and preventive schedule vs BTZ), while rats treated with the therapeutic schedule (that had received the first IVIg infusion at week 4) were not statistically different from BTZ-treated animals.

**Fig. 5:**
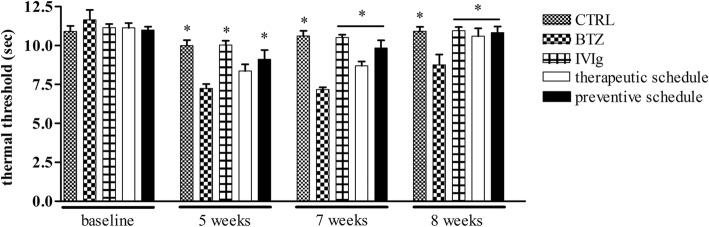
IVIg reduce BTZ-induced heat hyperalgesia. Paw withdrawal latencies of treated and control rats after BTZ treatment are shown. The protective effect of IVIg was observed in the preventive group already from the fifth week after the beginning of the study, while the therapeutic effect of IVIg appeared after 7 weeks (i.e., after the second IVIg infusion in this group). Data are reported as mean ± SEM. *p < 0.01 vs BTZ

After 7 weeks of treatment and until the end of the study, only the group treated with BTZ alone showed heat hyperalgesia (*p* < 0.01 all groups vs BTZ) (Fig. 5).

#### IVIg limits BTZ-induced reduction of IENF density

After 3 weeks, BTZ-treated groups had significant decrease of IENF density (*p* < 0.01 CTRL, IVIg vs BTZ).

**Fig. 6:**
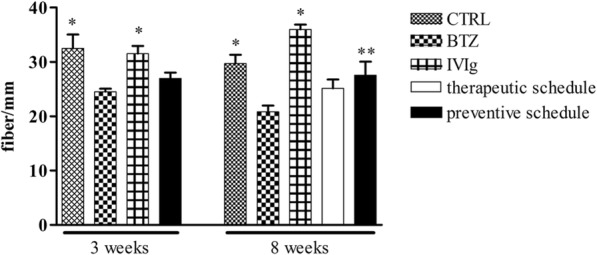
IVIg reduce the loss of innervation induced by BTZ. BTZ induced an evident reduction in the IENF density, which partially recovered at the end of co-treatment in the preventive group. Until week 3 from the beginning of the study, rats eventually belonging to the therapeutic schedule group are reported together with the animals of the BTZ group. Data are reported as mean ± SEM. *p < 0.01 vs BTZ; **p < 0.05 vs BTZ

At the end of treatment, the animals treated with BTZ alone and with the therapeutic IVIg schedule showed a statistically significant reduction in IENF density vs CTRL and IVIg group (*p* < 0.01 CTRL and IVIg vs BTZ). However, animals belonging to the preventive schedule group had a significant improvement of BTZ-induced changes (*p* < 0.05 preventive schedule vs BTZ) (Fig. 6).

#### IVIg reduces the severity of BTZ-induced axonopathy in peripheral nerves

At the end of treatment, the sciatic nerve analysis showed mild axonal changes in all the BTZ-treated rats, and since the overall extent of these changes was fairly limited, no clear effects of the administration of IVIg were observed.

**Fig. 7:**
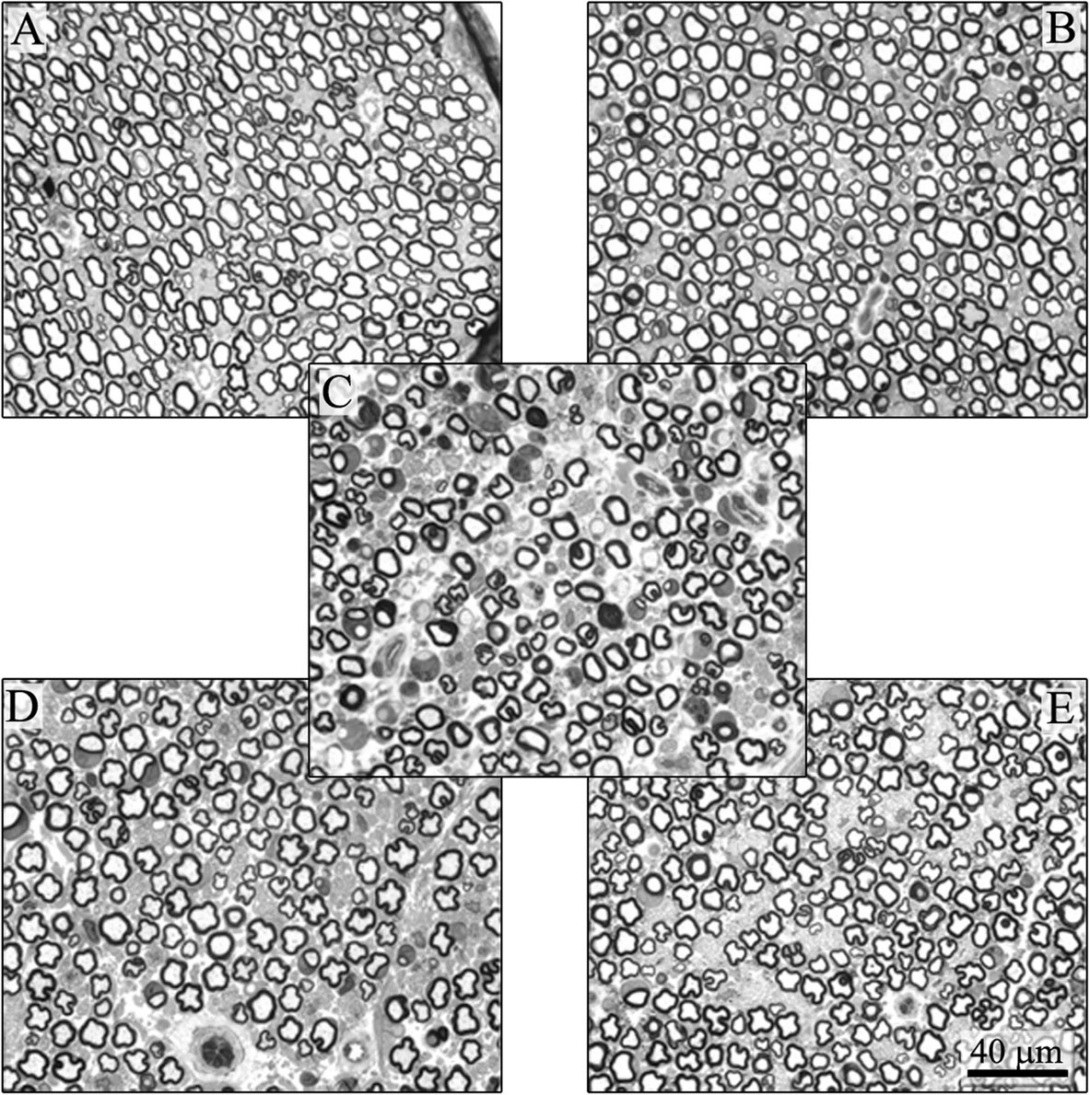
Effects of IVIg treatment on the caudal nerves of BTZ-treated rats. Severe axonopathy induced by BTZ administration was evident in the caudal nerve (**c**) if compared to controls (**a**) and IVIg-treated animals (**b**). The therapeutic administration of IVIg reduced the severity of the axonopathy (**d**), but the effect was more evident in the preventive setting (**e**)

However, more severe axonopathy (much more evident in the distal part of the nerves) and fiber density reduction were evident in caudal nerves collected at the end of treatment from rats treated with BTZ alone (Fig. 7c). The co-administration of IVIg in BTZ-treated rats reduced the severity of nerve fiber degeneration, particularly when delivered in the preventive setting (Fig. 7e). Co-treatment with IVIg both in preventive and therapeutic settings prevented BTZ-induced reduction in myelinated fibers diameter (end of treatment: CTRL mean ± SEM 7.05 μm ± 1.56; BTZ 6.62 μm ± 1.71; IVIg 7.19 μm ± 1.87; therapeutic schedule 6.91 μm ± 1.74; preventive schedule 7.18 μm ± 1.55; *p* < 0.01 all groups vs BTZ). Analyzing the histograms of the myelinated fiber size distribution, it was confirmed that fiber loss involved mainly medium and large caliber fibers in the group treated with BTZ alone, while this selective loss was not present in the animals treated with both schedules of IVIg (data not shown).

#### IVIg suppresses macrophage infiltration in peripheral nerves

**Fig. 8:**
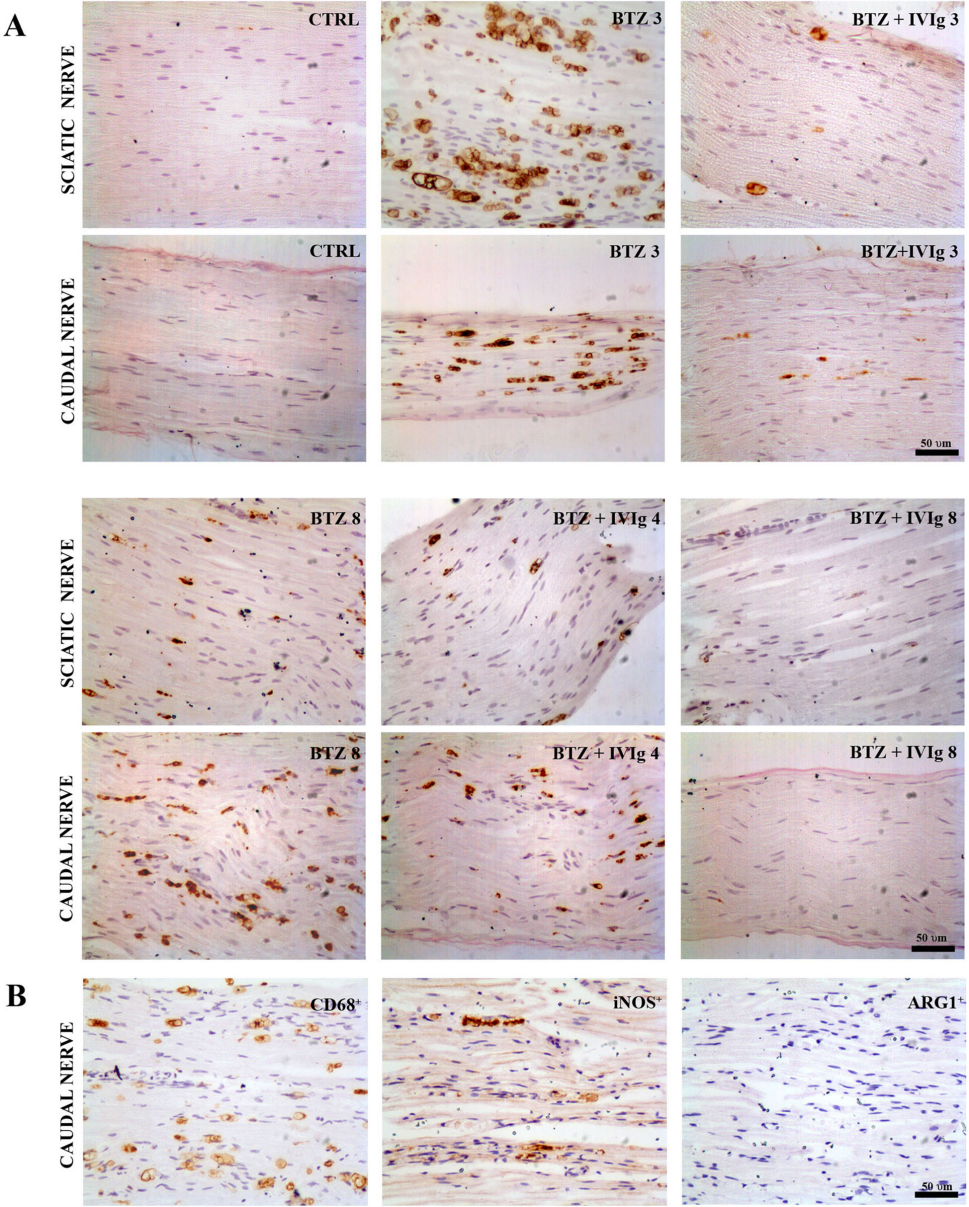
Effects of IVIg treatment in inflammatory infiltration in peripheral nerves. **a** Immunohistochemistry for CD68: After 3 and 8 weeks of BTZ treatment, both sciatic and caudal nerves show a massive macrophage infiltration which is almost completely abrogated by the preventive co-treatment with IVIg (BTZ + IVIg 3; BTZ + IVIg 8). In the therapeutic setting (BTZ + IVIg 4), a milder reduction of CD68^+^ infiltrating cells was achieved in both sciatic and caudal nerves. Scale bar 50 μm. **b** Representative immunohistochemistry for iNOS and ARG1 after 3 weeks of BTZ treatment. Most infiltrating macrophages (CD68+) are iNOS^+^ pro-inflammatory M1 type while a very limited amount of them are ARG1+ anti-inflammatory M2 type. Scale bar 50 μm

IHC for CD68 evidenced a robust macrophage infiltration in sciatic and caudal nerves as early as after 3 weeks of BTZ administration. The co-administration of IVIg for 3 weeks in the preventive setting remarkably reduced the number CD68^+^ cells. After 8 weeks of BTZ treatment, the infiltration of CD68+ cells in sciatic and caudal nerves persisted. The preventive schedule caused a nearly complete abolition of infiltration (Fig. 8a).

To further characterize infiltrating CD68^+^ cells, we performed an IHC for iNOS (pro-inflammatory macrophages) and for ARG1 (anti-inflammatory macrophages) on adjacent nerve slides of BTZ-treated rats, evidencing that nearly all infiltrating cells were iNOS positive at 3 weeks (Fig. 8b). Similar results have been obtained at 8 weeks (data not reported).

Semi-quantitative analysis on anti-CD68 stained sections confirmed the morphological observations. Table 1 shows that the co-treatment of BTZ with IVIg for 3 weeks significantly reduced the amount of CD68^+^ cells respect to BTZ-treated. In the specimens collected at the end of the study, no clear reduction in CD68^+^ cells was observed after the injection of IVIg in the therapeutic schedule while a remarkable reduction of CD68^+^ infiltrating cells was evidenced in the preventive schedule.

**Table 1:** Semi-quantitative analysis of CD68^+^ infiltrating cells

Samples obtained 3 weeks from the beginning of the study	Caudal nerve
CTRL	+
BTZ	+++
IVIg	neg
Preventive schedule	+
Samples obtained at the end of the study (8 weeks)	Caudal nerve
CTRL	neg
BTZ	+++
IVIg	neg
Therapeutic schedule	+++
Preventive schedule	+

+ 1–5 macrophages/section; ++ 5–30 macrophages/section; +++ > 30 macrophages/section

## Discussion

IVIg are an accepted and effective treatment in immuno-mediated chronic inflammatory neuropathies such as chronic inflammatory demyelinating polyradiculoneuropathy (CIDP) and multifocal motor neuropathy (MMN), and they are also effective in reducing neuropathic pain in the acute forms [23].

In this study, we used a human formulation of immunoglobulins in our rat model of BIPN. IVIg doses were in the range of the clinical schedules. Although human IVIg have already been used in rodent models [24, 25], their schedules were not based on solid evidence and, therefore, we performed a preliminary pilot study to determine the PK parameters needed to select a suitable schedule and to test safety and tolerability of IVIg.

Our study evidenced that IVIg were effective particularly when administered in a preventive setting (i.e., from the beginning of BTZ administration), but a significant effect on drug-induced allodynia was also present using IVIg after 4 weeks of BTZ treatment, when allodynia was already present. The pathologic correlate of this observation was the significant, although mild, prevention of BTZ-induced IENF density reduction achieved in the group treated with the preventive schedule, and this predominant effect on small fibers could explain the absence of significant neurophysiological-evident protection since it would be mainly related to large myelinated fiber activity. An alternative explanation for the apparent discrepancy between neurophysiological and pathological results induced by IVIg administration might be that since BTZ induced axonopathy (i.e., an event less rapid to be reverted at the functional level than primary demyelination), the protective effect of IVIg is not yet captured by the neurophysiological examination, although clearly evident at the direct examination of the nerves.

The effect of IVIg administration on small nerve fibers was also associated with pathological evidence of reduced axonal damage in peripheral nerves, again more pronounced using the preventive schedule of administration. This was particularly evident in the distal part of the caudal nerve, in line with the typical BIPN pathological features of distal axonopathy [21].

It is remarkable that changes in the peripheral nerves were associated with a clear reduction in macrophage infiltration, an event already present after 3 weeks of BTZ administration in association with the earliest signs of nerve damage. Since the vast majority of infiltrating cells were macrophages with pro-inflammatory phenotype and IVIg nearly completely abrogated this pathologic event, it is reasonable to hypothesize that the protective effect of IVIg in BIPN is related to immunomodulation, a well-established effect of their clinical use. In agreement to our hypothesis, Tzekova and colleagues have demonstrated in Schwann cell cultures that IVIg induce axonal outgrowth by secreting interleukin-18 (IL-18) [26], a proinflammatory cytokine transiently induced after peripheral nerve injury [27].

Neuroinflammation has been reported to be a relevant pathogenetic event in several peripheral nervous system diseases, particularly when neuropathic pain is a prominent feature [28] and it occurs in several forms of CIPN. Antitumor drugs target both immune system and microglia, and they are able to induce increased migration of macrophages and release of proinflammatory cytokines [29]. It is known that these mediators act sensitizing nociceptive signaling in peripheral and central nervous systems, suggesting an immune pathogenesis for neuropathic pain [30].

The interaction between neuroimmune activation and the involvement of glial cells in the onset and maintenance of chronic pain has been the subject of increasing research over the last two decades [31]. Specifically regarding CIPN, PTX-induced mechanical allodynia has been associated with infiltration of macrophages into DRG [32], increased expression of tumor necrosis factor-alpha and interlukin1, beta (TNF-α, IL1-β), associated with suppression of interlukin-10 (IL-10) and interlukin-4 (IL-4) in the spinal cord [33], as well as with induction of monocyte chemoattractant protein-1 (MCP-1) and its receptor CCR2 in DRG [34]. Accordingly, in the spinal cord of PTX-treated animals, activation of astrocytes and increased levels of several inflammatory cytokines and chemokines, such as TNF-α, interferon-γ (INFγ), CCL11, CCL4, CCL3, IL-12p70, and GM-CSF, were observed [35]. Astrocyte activation in the spinal cord has also been suggested to contribute to oxaliplatin and bortezomib-induced nocifensive behaviour in rats [36, 37].

Further support to a possible role of neuroinflammation in CIPN has recently been provided investigating the role of the innate immunity mediator Nod-Like Receptor Protein 3 (NLRP3) Inflammasome. Inflammasome activation cleaves caspase-1, inducing secretion of proinflammatory cytokines (IL1β and IL-18) and cell death. Consistent evidence suggests increased expression of NLRP3 inflammasome in macrophages infiltrating DRG and sciatic nerves, and this might contribute to the development of neuropathic pain in short-term PTX-treated rats [38]. It is also remarkable that inhibition of NLRP3-mediated inflammatory response in the spinal cord results in the attenuation of mechanical hypersensitivity in mouse models of neuropathic pain, underscoring the link between pain and inflammation [39] Similar data have also very recently been reported also following bortezomib administration using an acute (five consecutive days) experimental setting in rats [40].

Our chronic BIPN animal model is an ideal target to further investigate if neuroinflammation might be a therapeutic target to reduce the severity of anticancer drugs-induced peripheral neurotoxicity in clinical setting, since it is characterized by severe neuropathic pain, associated with evident pathological changes such as axonal nerve damage and reduction in IENF density.

## Conclusions

Our results not only suggest a prominent role of neuroinflammation in BIPN, but also indicate the need for its further investigation in other forms of chronic CIPN where neuropathic pain has different features and manifestations.

Moreover, these results also support the use of IVIg as a safe and possible treatment option against BIPN.

## Abbreviations

CIPN: Chemotherapy-induced peripheral neurotoxicity
BIPN: Bortezomib-induced painful neuropathy
IVIg: Intravenous immunoglobulins
DRG: Dorsal root ganglia
IENF: Intraepidermal nerve fiber
